# Predictive Markers of Efficacy for an Angiopoietin-2 Targeting Therapeutic in Xenograft Models

**DOI:** 10.1371/journal.pone.0080132

**Published:** 2013-11-14

**Authors:** Gallen Triana-Baltzer, Adam Pavlicek, Ariadne Goulart, Hanhua Huang, Steven Pirie-Shepherd, Nancy Levin

**Affiliations:** 1 CovX Research, Pfizer Worldwide Research and Development, San Diego, California, United States of America; 2 Oncology Research Unit, Pfizer Worldwide Research and Development, San Diego, California, United States of America; Northwestern University Feinberg School of Medicine, United States of America

## Abstract

The clinical efficacy of anti-angiogenic therapies has been difficult to predict, and biomarkers that can predict responsiveness are sorely needed in this era of personalized medicine. CVX-060 is an angiopoietin-2 (Ang2) targeting therapeutic, consisting of two peptides that bind Ang2 with high affinity and specificity, covalently fused to a scaffold antibody. In order to optimize the use of this compound in the clinic the construction of a predictive model is described, based on the efficacy of CVX-060 in 13 cell line and 2 patient-derived xenograft models. Pretreatment size tumors from each of the models were profiled for the levels of 27 protein markers of angiogenesis, SNP haplotype in 5 angiogenesis genes, and somatic mutation status for 11 genes implicated in tumor growth and/or vascularization. CVX-060 efficacy was determined as tumor growth inhibition (TGI%) at termination of each study. A predictive statistical model was constructed based on the correlation of these efficacy data with the marker profiles, and the model was subsequently tested by prospective analysis in 11 additional models. The results reveal a range of CVX-060 efficacy in xenograft models of diverse tissue types (0-64% TGI, median = 27%) and define a subset of 3 proteins (Ang1, EGF, Emmprin), the levels of which may be predictive of TGI by Ang2 blockade. The direction of the associations is such that better efficacy correlates with high levels of target and low levels of compensatory/antagonizing molecules. This effort has revealed a set of candidate predictive markers for CVX-060 efficacy that will be further evaluated in ongoing clinical trials.

## Introduction

The process of angiogenesis in neoplastic development can be carried out by a large number of angiogenic activators including VEGF (vascular endothelial growth factor), MMPs (matrix metaloproteases), PIGF (placental growth factor), FGFs (fibroblast growth factors), HGF (hepatocyte growth factor), PDGFs (platelet-derived growth factors), and Ang family proteins (angiopoietins). Compounds targeting these factors (or their cognate receptors) have shown anti-angiogenic effect and often significant and pan-tumor inhibition in preclinical models. In 2003 the first anti-angiogenic agent obtained FDA approval (bevacizumab) and several other agents have followed suit in the past decade. In general this class of agents has shown substantial benefit in a wide variety of cancers yet has been plagued by three primary issues: acquired resistance, rebound of tumor growth upon withdrawal of compound, and lack of biomarkers to predict or track response [1]. The latter point is of special note as not all patients in any given indication show response and the potential toxicities with particularly VEGF-targeting agents highlights the need to prospectively identify patients that are unlikely to benefit from these therapies. Despite over a decade of intense preclinical and clinical evaluation no single marker/method that consistently predicts responsiveness for an anti-angiogenic agent has been identified [2-4].

In contrast to the depth of preclinical knowledge, clinical experience, FDA-approved compounds, and reported biomarker work on the VEGF pathway, much less is known about targeting the angiopoietin pathway. VEGF and angiopoietins are thought to act at different times and with different roles in the angiogenic process; VEGF directs vascular sprouting, while angiopoietins facilitate vascular maturation and remodeling [5-8]. Angiopoietins are released by tumor cells and endothelial cells (TCs and ECs, respectively) and are thought to act primarily on the ECs via interactions with the Tie2 receptor and integrins [9-12]. In the context of stimulation of the Tie2 receptor on ECs Ang1 is thought to be the primary agonist [13] leading to pro-maturation signaling via EC survival [14,15] and recruitment of pericytes to seal the vessels [16,17]. Ang2 on the other hand is believed to be a much weaker agonist of Tie2, and as a competitor with Ang1 functionally causes a reduction in Tie2 agonism, leading to pro-remodeling signaling [18-21]. The process of destabilizing vasculature to allow for remodeling, via loss of pericytes and ECs, is believed to be an initial step in formation of new blood vessels. Therapeutic inhibition of Ang2 signaling should theoretically lead to stronger Ang1 signaling and hence vascular stabilization. Indeed the compound CVX-060 (PF-04856884), an Ang2-specific trap, induces significant reductions in microvessel density and tumor growth in several preclinical models [22].

CVX-060 has shown promising efficacy and limited toxicity in a phase 1 trial (NCT00879684), and is now being tested in a phase 2 RCC trial (NCT01441414) [23,24]. As no information exists regarding predictive biomarkers for angiopoietin therapy we performed a systematic study to identify biomarkers correlating with CVX-060 efficacy in xenograft (XG) models. Here we describe the design of a promising campaign to identify biomarkers for anti-angiogenic agents in preclinical models and report a multi-protein signature that correlates with tumor growth inhibition (TGI) by CVX-060 across a wide variety of tumor types. An initial set of XGmodels were used to build a predictive model, and then a second set of XG models were used for an independent and blinded prospective evaluation of the model. The results indicated that higher Ang2 and lower Ang1 may correlate with better CVX-060 response, consistent with known angiopoietin biology. In addition lower EGF/EGFR and Emmprin may correlate with better CVX-060 response. Using only Ang1, EGF, and Emmprin as a multi-protein signature yielded a predictive model with considerable accuracy in the prospective evaluation.

## Materials & Methods

### Tumor xenograft studies

Cell lines (ATCC, Manassas, VA; or TGEN, Scottsdale, AZ) were cultured as suggested by their commercial source to subconfluence, harvested with trypsin, and mixed 1:1 with Matrigel (BD Bioscience) immediately before implantation in the upper right flank of young adult female *Nu-Foxn1* ^*nu*^ mice (Charles River Laboratories, Wilmington, MA; or Harlan, Indianapolis, IN) (except SKOV3 which was implanted in NOD.Cg-*Prkdc* ^*scid*^ *Il2rgtm1Wjl*/SzJ (NSG) mice). Patient-derived XG (PDX) models (OVX243 and OVX276) were passaged in vivo to achieve sufficient material for dosing studies, and were used for the CVX-060 efficacy study at 5-6 passages after collection from human tumor. Tumor explants were implanted in the upper right flank of young adult female *Nu-Foxn1* ^*nu*^ mice. Tumor volume was measured twice weekly using the formula: Volume = (Length x Width^2^) x π/6. Subcutaneously inoculated tumors staged to the desired volume (average about 350 mm^3^) were randomized and dosed accordingly. Ten animals were used per group: Group 1 = vehicle (PBS), Group 2 = CVX-060 (produced at Pfizer, [22]) at 10 mg/kg, Group 3 = no treatment. Group 3 was allowed to grow to average of 500 mm^3^ before collecting for “pretreatment size” profiling. Group 1 and 2 were dosed intraperitoneally in a volume of 0.2 mL per mouse once per week (i.p. QW) until vehicle group mean reached ~2000 mm^3^ at which time all groups were terminated. Tumor growth inhibition (TGI) was determined as %TGI = (1-treatment growth/control growth) x 100 at termination. 

### Ethics statement

Tumor tissues for PDX studies were obtained from patients in Hebei Medical University Fourth Hospital through collaboration with Beijing Keluoen Translational Medicine Institute with approval by the Institutional Review Boards of the hospital and the written informed consent from patients. All animal procedures conducted in China at Crown Bioscience SPF facility were in strict accordance with the Guide for the Care and Use of Laboratory Animals of the National Institutes of Health. The protocol was approved by the Committee on the Ethics of Animal Experiments of Crown Bioscience (Crown Bioscience IACUC Committee). All other animal experiments were conducted under the institutional guidelines of CovX/Pfizer, TGEN Drug Development, Jackson Laboratory, or Crown Bioscience’s Institutional Animal Care and Use Committee. CovX is an AALAC-accredited unit (#001442). 

### Tumor collection and sample preparation

Pretreatment size tumors were collected at ~500 mm^3^, immediately bisected and flash frozen and stored at -80°C until analysis. For nucleic acid extraction tumors were homogenized in FastPrep microhomogenizers with lysing matrix D (MP Biomedicals, Pasadena, CA) using 1 mL trizol/chloroform method (Invitrogen, Chicago IL), followed by isolation with DNeasy spin columns (Qiagen, Valencia, CA). Nucleic acid concentration was determined by UV, followed by dilution to equivalent levels and storage at -80°C until analysis. For protein extraction tumors were homogenized in FastPrep microhomogenizers with lysing matrix D using 1x protease/phosphatase inhibitor cocktail (HALT, Thermo Scientific, Waltham, MA), followed by addition of lysis buffer (Cell Signaling Technology, Beverly, MA) and incubation at 4°C for 1 hr. Lysates were clarified by centrifugation (14000 rpm, 10 min, 4°C) and supernatants were used directly for ELISAs.

### Somatic mutation analysis

Mutation status for genes of interest was determined via comparison with published data where possible, and via mass spectrometry analysis of 238 somatic mutations across common oncogenes in genomic DNA from The OncoCarta Panel v1.0 (Sequenom, San Diego, CA) for confirmation. 

### Snp analysis

Single nucleotide polymorphism (SNP) status for the locations of interest was determined via qPCR of genomic DNA using specific primer/probes to the exact SNP locations (rs699947, rs833061, rs1570360, rs2010463, rs3025039, rs3814055, rs11549467, rs4073; Applied Biosystems, Carlsbad, CA). SNP haplotype was determined by observing relative Ct for each nucleotide possibility.

### ELISA

Tumor lysate was analyzed for specific protein maker levels using ELISA kits predesigned and validated for each marker (R&D, Minneapolis, MN). Total protein concentration in the lysates was determined via BCA method (Thermo Scientific). Marker concentration in each individual sample was then normalized to the total protein concentration in the same sample for a ng marker/g total protein value. Values shown for each XG model represent the median concentration from 3-10 tumors per model for 88% of the analyses, the remaining 12% of the data employed less than 3 tumors per model/marker. 

### Data Analysis

Replicates of xenograft TGI and protein profiling experiments were summarized using median values and are available in Table S1. Comparison between TGI and linear baseline protein expression values was performed using the Pearson linear correlation coefficient implemented in R. The model for prediction of TGI values was constructed using BRB-ArrayTools v4.2.1 [25]. The linear protein expression values were then transformed into log_2_ values. To develop the classifier on the training data, we applied the quantitative trait prediction workflow in BRB-ArrayTools, using Least Angle Regression (LAR) [26] with default parameters: 0 % error threshold, no inclusion of 2 way interactions, and 10-fold cross-validation. The full LAR model with all parameters is available in Figure S1.

## Results

### CVX-060 induced TGI in “training set” xenograft models

In order to evaluate the commonality of CVX-060 efficacy a panel of 15 cell line and patient derived XG models were dosed with vehicle or CVX-060 at 10 mg/kg QW. To potentially limit variability between indications we focused on models from 3 tumor types; ovarian cancer, renal cell carcinoma, and colorectal cancer. Tumor growth curves and calculated tumor growth inhibition at termination revealed a wide range of CVX-060 responses (0-64% TGI, median = 27%), with no preference for tumor type (Figure S2 and Table 1). A cohort of pretreatment size tumors (500 mm^3^) were collected from each study for profiling in order to identify predictive biomarkers of CVX-060 affect. Correlations of somatic mutations, single nucleotide polymorphisms (SNPs), or protein level with CVX-060 induced TGI were then evaluated.

**Table 1:** CVX-060 induced tumor growth inhibition in “training set” xenograft models.

model	type	median CVX-060 TGI%
OVCAR5	ovarian	0
ES2	ovarian	11
HeyC2	ovarian	21
IGROV1	ovarian	23
A2780	ovarian	32
OVX276	ovarian*	38
SKOV3	ovarian	49
OV90	ovarian	51
OVX243	ovarian*	61
A498	RCC	22
Caki1	RCC	26
G401	RCC	32
SN12CCP	RCC	45
HT29	CRC	27
Colo205	CRC	64

* patient-derived xenograft

### Lack of correlation between TGI and common somatic mutations or angiogenesis-related SNPs

Somatic mutations in the VHL, SetD2, and VEGFR2 genes have been observed in human tumors and implicated in response to hypoxia and potentially anti-angiogenic compounds [27-34]. Numerous other more commonly mutated genes are also used to segregate patients and predict sensitivity to marketed drugs (BRAF, KRAS, EGFR, etc.) [35,36] and may play a role in general response to anti-angiogenic compounds. We therefore profiled for mutation status in a set of 11 genes and looked for enrichment of any of these mutations in the highly responsive or less responsive XG models. Mutation status did not correlate with TGI for any of the 11 genes evaluated (Table S2).

Several VEGF gene SNP haplotypes have been reported to correlate with particular anti-angiogenic response in retrospective analysis of clinical trials [37,38]. Additional SNPs in NR_1/2_, Hif1a, and IL8 have similarly been implicated in anti-angiogenic response in clinic [38]. We therefore profiled for SNP status at 8 discrete regions suggested from literature and looked for enrichment of any of these SNP haplotypes in the highly responsive or less responsive XG models. SNP status did not correlate with CVX-060 TGI for any of the 8 SNPs evaluated (Table S3). A similar analysis was performed with TGI values from a VEGF-targeting therapeutic which suggested correlation between TGI and several of these SNP haplotypes (data not shown), indicating potential for SNP predictive power in XGs.

### Correlation between subset of pretreatment marker levels and CVX-060 effect

Levels of specific proteins in serum or tumor are commonly used as predictive or pharmacodynamic markers in clinic. A meta-analysis of literature on preclinical VEGF-therapy resistance mechanisms and correlates of clinical VEGF-therapy induced OS/PFS yielded a list of 22 markers of potential general relevance to anti-angiogenic compounds [2-4,39-43]. Additionally a differential gene expression analysis previously performed on Colo205XG tumors acutely treated with vehicle or CVX-060 identified additional pharmacodynamic markers potentially involved in CVX-060 response (data not shown). A list of all 27 markers evaluated is shown in Table 2.

**Table 2:** Pearson’s correlation of marker concentration as quantified by ELISA vs. Median CVX-060 TGI%.

**Protein**	**Pearson's R**	**p-value**
EGFR	-0.55	0.07
EMMPRIN	-0.50	0.06
Angpt1	-0.44	0.10
EGF	-0.40	0.14
cMET	-0.28	0.31
FGF2	-0.27	0.35
Angplt4	-0.26	0.35
Axl	-0.22	0.43
GCSF	-0.20	0.47
MMP7	-0.17	0.59
THBS1	-0.15	0.58
MCP1	-0.15	0.62
PIGF	-0.14	0.63
PDGFRb	-0.13	0.63
huSDF1a	-0.13	0.69
PDGFRa	-0.03	0.92
VEGF	-0.03	0.93
HGF	-0.01	0.98
PROK1	0.03	0.92
TGFa	0.09	0.75
IL8	0.11	0.73
Hif1a	0.18	0.59
pmTOR	0.19	0.53
msSDF1a	0.25	0.43
PDGFbb	0.31	0.35
Angpt2	0.41	0.13
Hif2a	0.42	0.26

**Figure 1:**
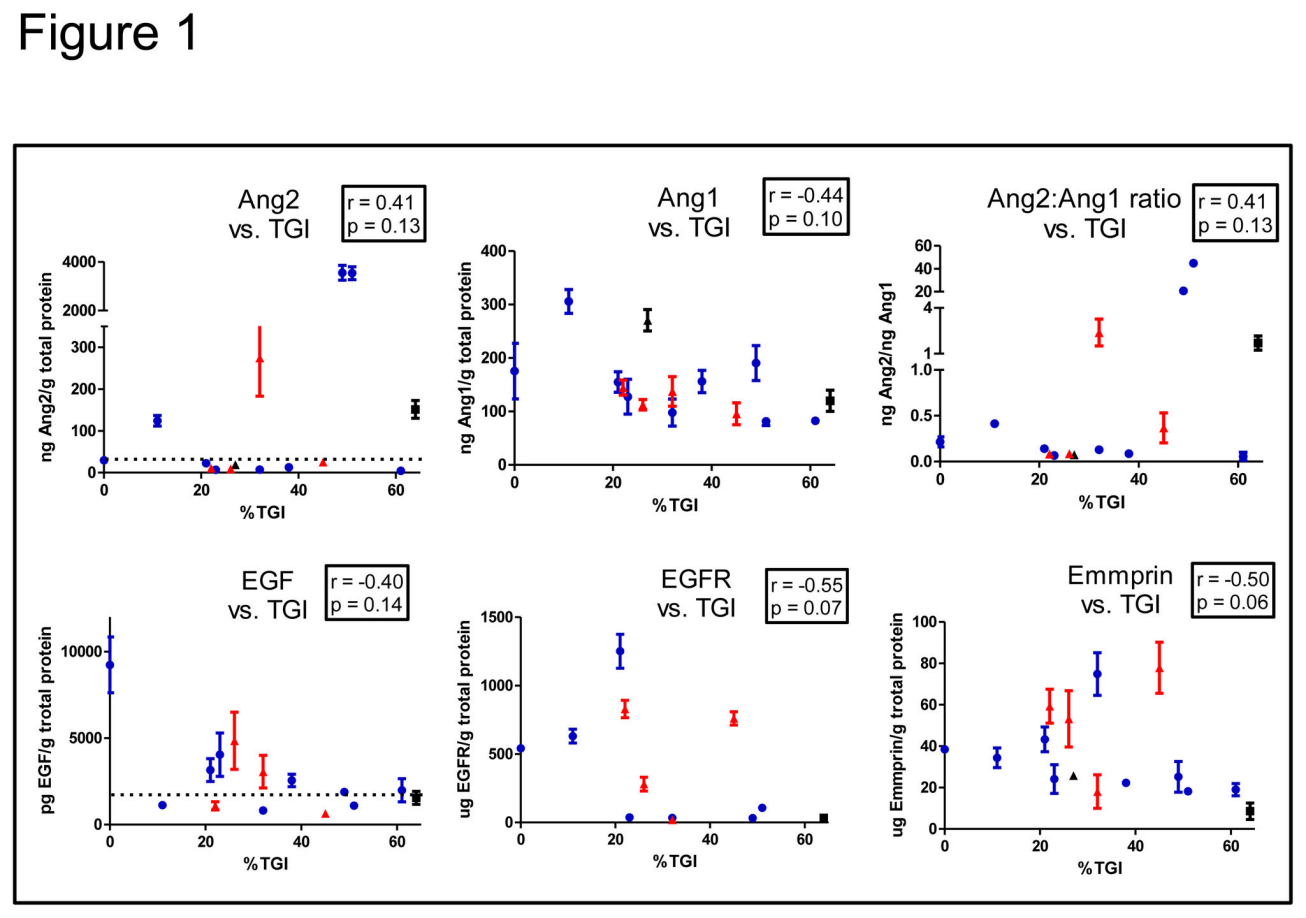
Correlation between pretreatment marker levels and CVX-060 effect. Representative examples from Table 2 are graphically shown to illustrate the relationship between marker level (mean +/- SEM) and median CVX-060 TGI (%) in training set models. Each dot represents a single XG or PDX model, color coded by tumor type: blue = ovarian, red = RCC, black = CRC. Dashed line indicates lower limit of quantification. r = Pearson’s coefficient, p = p-value.

Pretreatment tumors from the “training set” XG models were subjected to ELISA to quantify the levels of each of the 27 protein markers of interest. Alignment of median marker level of each vs. median CVX-060 TGI revealed that higher Ang2 tracked with higher TGI (Pearson R = 0.41, n.s.), as predicted given this is the target of CVX-060. Similarly lower Ang1 (Pearson R = -0.44, n.s.), and hence higher Ang2:1 ratio (Pearson R = 0.41, n.s.), tracked with higher TGI. Finally lower EGF, EGFR, and Emmprin each tracked with higher TGI (Figure 1). These findings did not appear to be restricted to any one tumor type (e.g. ovarian, RCC, or CRC) as representative models from each type could be found along the curve in each graph.

Pearson’s R values and p-values of all measured proteins are listed in Table 2. Despite trending with response, none of the markers used independently were significantly associated with response at the significance level of 5% (p<0.05). However due to small sample size this might be expected or a multi-protein signature may be more useful. Setting a lower significance bar (p< 0.2) indicated that amongst all the markers evaluated the levels of Ang2, Ang1, EGF, EGFR, and Emmprin each had the greatest correlation with CVX-060 TGI. These markers were prospectively used to derive a new predictive model described in the next section.

### Generation and testing of predictive marker model

**Figure 2:**
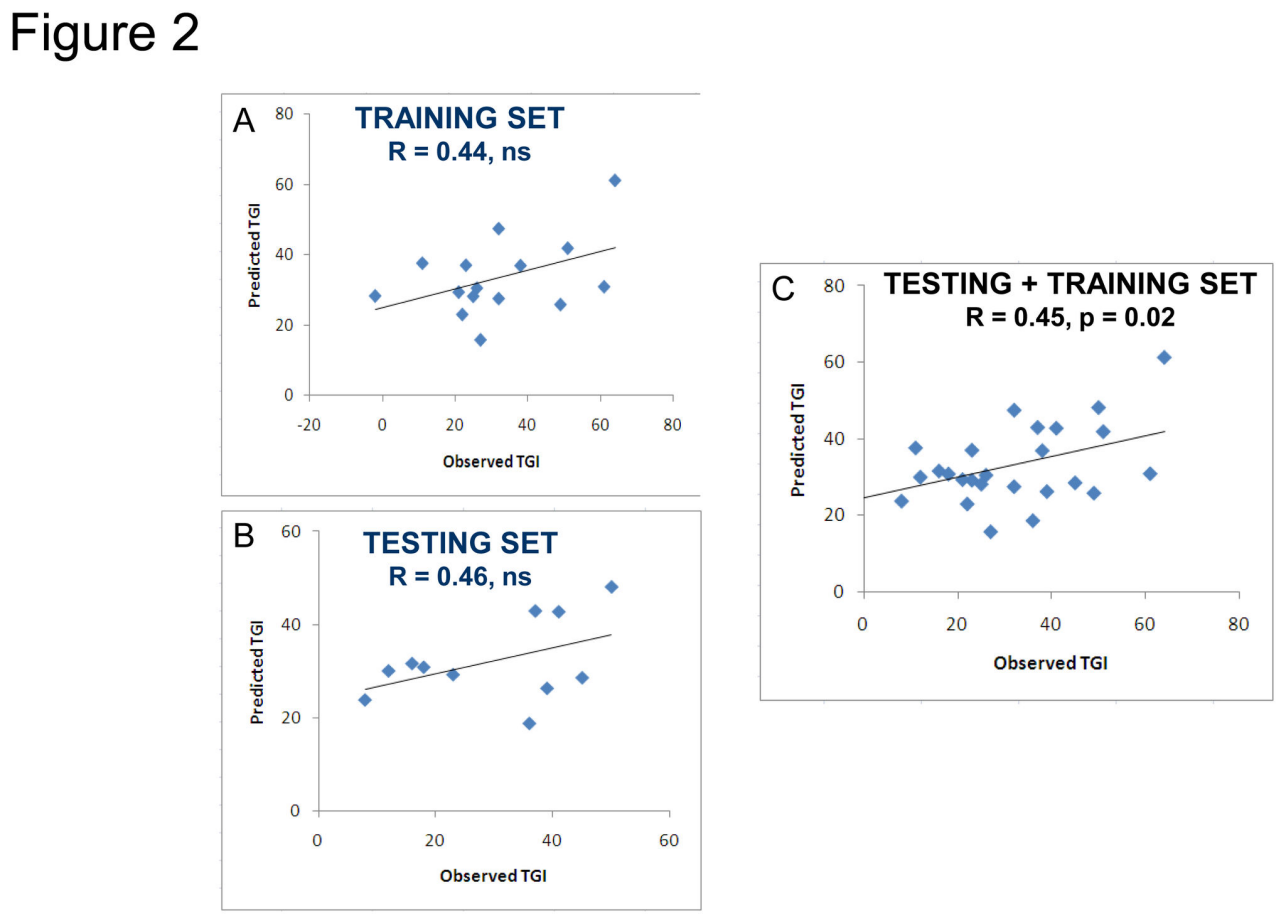
A three-protein model for prediction of TGI. Least Angle Regression (LAR) method identified 3 markers (Ang1, EGF, & Emmprin) sufficient to build a model to predict CVX-060 TGI. Model performance in the training set (A) and testing set (B) of xenograft lines is shown. Statistical significance was not achieved until combining both sets (C). Each dot represents the model predicted TGI% vs. median observed TGI% of a single xenograft line.

We have used Least Angle Regression (LAR) available in BRR-ArrayTools to develop a new multiprotein signature of response to CVX-060 in vivo. LAR is useful in predicting quantitative traits when the number of independent variable (genes or protein) is larger than the number of analyzed samples [25,26]. A model was constructed to identify the minimal number of markers necessary for predictive power using the 15 XG models from the training set and the 5 TGI-correlated proteins specified above. This exercise yielded a set of 3 markers: Ang1, EGF, and Emmprin. The LAR model composed of 3 markers was used to generate a predictive model that in the training set predicted TGI values correlated with observed TGI values (R = 0.44, n.s.) (Figure 2A). 

The ultimate test of any predictive biomarker/s is a prospective evaluation in new models. Towards this end we chose 11 additional XG models as a “testing set” (based on gene expression analysis identifying these models as possessing extreme levels of the key markers) and measured protein expression of the markers of interest in pretreatment size tumors. Protein levels of Ang1, EGF, and Emmprin were then used to predict TGIs, followed by running efficacy studies with CVX-060 in each model. 

A comparison of predicted TGI values from the 3-protein regression model with the observed TGI values in the testing set XGs yielded comparable correlation to that seen in the training set XGs (R = 0.44, n.s., Figure 2B). While the correlation between predicted and observed TGIs was suggestive but not statistically significant in both sets, the combination of both sets yielded a significant correlation between predicted and observed values (R = 0.45, p = 0.02, Figure 2C). A comparison of predicted and observed TGIs indicated that 6/11 models were correctly predicted within 15% TGI (15% being used as a cutoff based on common experimental variability with TGI quantification) (Table 3). Taken together the prospective analysis indicated that the 3-protein set of Ang1, EGF, and Emmprin may have utility in predicting response to CVX-060.

**Table 3:** Prospective testing of the predictive marker hypothesis.

model	type	Ang2 ng/g	Ang1 ng/g	VEGF ng/g	EGF ng/g	EGFR µg/g	Emmprin µg/g	Predicted TGI%	Observed TGI%	Prediction within 15% of observed?
U251	GBM	5	293	15719	5.8	26	99	19	**36**	**N**
NCI-H441	NSCLC	20	91	2876	9.9	13	67	24	**8**	**N**
D54MG	GBM	5	14	607	3.8	3	102	26	**39**	**Y**
NCI-H720	NSCLC	6907	266	305	2.9	14	34	29	**45**	**N**
SKMEL1	melanoma	3948	483	30	11.1	3	21	29	**23**	**Y**
A431	melanoma	41	56	3392	2.6	361	46	30	**12**	**N**
NCI-H209	SCLC	1966	258	695	2.4	9	27	31	**18**	**Y**
SHP-77	SCLC	3331	164	365	1.2	10	31	32	**16**	**N**
MDA-MB435	Breast/melanoma	8	16	552	2.8	1	13	43	**41**	**Y**
U87	GBM	6	14	263	0.8	3	17	43	**37**	**Y**
A549	NSCLC	4	28	124	6.3	1	5	48	**50**	**Y**

Marker levels shown in median ng target/g total protein in untreated 500 mm^3^ tumors (n = 2-10), except for EGFR and Emmprin which are shown at µg/g levels. CVX-060 dosed and TGI% defined as in Table 1.

## Discussion

Biomarkers predicting clinical antiangiogenic therapy response have proved elusive, yet are sorely needed due to lack of consistent response of the therapeutic class in any given oncological indication. While clearly not perfect mirrors of the clinical situation animal models of cancer are useful tools for building and validating predictive hypotheses, particularly for anti-angiogenic agents which usually only show (indirect) anti-tumor cell effect in vivo. Building and testing predictive hypotheses however often requires large number of patients/models and so the cost, time, and labor involved in doing this entirely with in vivo models is daunting and without rigor can yield no significant findings. Here we describe an attempt to address this problem and in doing so reveal a multi-protein signature that may be predictive of anti-Ang2 therapy response in animal models.

CVX-060 has been well tolerated and exhibited promising efficacy in a phase 1 trial (NCT00879684) [23,24]. As CVX-060 is the first clinical compound to specifically target Ang2 the potential for identifying biomarkers is entirely unknown. Indeed at this time there are no published reports of biomarkers for any angiopoietin targeting compounds. The results described here suggest that the levels of 3 specific tumor proteins (Ang1, EGF, Emmprin) taken together as a multi-expression signature may be predictive of anti-Ang2 therapy in XG models. As confirmation of the findings the direction of each markers correlation with CVX-060 response is intuitive: higher levels of the CVX-060 target Ang2 correlated with better response, and lower levels of all the other markers correlated with better response. Ang1 and Ang2 are thought to compete for binding to the signaling receptor Tie2, thus lower levels of Ang1 would allow Tie2 signaling to be predominantly driven by Ang2 hence pre-establishing a enhanced sensitivity to Ang2-therapy. EGF/EGFR and Emmprin are known to have roles in angiogenesis and VEGF-therapy response [44-49], and so could be considered as compensatory pathways to circumvent angiopoietin therapy-induced hypoxia. 

The lack of statistical significance seen with the individual biomarkers in the training set, or the 3-marker signature in the testing set, may be explained by small sample size. Indeed the findings themselves are intuitive as suggested above, and the combination of training and testing sets allowed for statistical significance with the 3-marker signature. However, as statistical significance is lacking in the blinded analyses the conclusions presented here should be taken with caution and require further evaluation. 

It is unclear if the predictive power of this multi-expression signature will hold true for other anti-angiopoietin compounds that are not as specific for Ang2 as CVX-060 [22]. In particular these results indicate that lower Ang1 levels would correlate with better CVX-060 affect, however compounds that target both Ang1 and Ang2 might theoretically work better with high levels of both targets. 

All assays used here to detect somatic mutations, SNPs, and proteins were designed to be human specific in order to focus on variable provided by the implanted tumor, rather than the mouse host, and so can be described as being tumor derived rather than endothelial, stromal, or serum components. While this approach would not detect any of the myriad of supporting cell components involved in tuning angiogenesis/hypoxia detection we felt that a focused approach was needed in order to tease out any significant correlations within the complex choreography of angiogenesis. By examining only tumor expressed components in effect this study is designed to capture importance of target and Tie2-competitor ligands (Angiopoietins) and the tumors response to the downstream hypoxia induced by vascular collapse. In this study there is no evaluation of the intermediate step in CVX-060 therapy, the endothelial cell response to Ang2 deprivation, and indeed some evidence indicates that variation in the endothelial proteome can impact Ang2 function [10].

It should be noted that the work done here employed a large number of tumor types (multiple models of RCC, Ovarian, NSCLC, SCLC, GBM, and melanoma) and thus the multi-expression signature can be defined as a pan-tumor predictive model. Despite the fact that the training and testing cohorts employed different tumors types the correlation between predicted and observed TGI values was similar in the both sets. Typically one would desire to work with more homogeneous sets in biomarker work, in particular by working entirely within one tumor type, however due to requiring a minimal sample size for statistics this may not often be possible using XGs. By building our training set in RCC and Ovarian models and then switching to other tumor types for the validation set the bar for predictive performance was potentially higher. This suggests that the multi-expression signature we define here could be pan-tumor in nature and/or that enrichment in a specific tumor type could improve the predictive power of this model.

Beyond the results of this work an examination of the study design employed could be useful for guiding future biomarker discovery. To search for biomarkers we first ran a differential gene expression screen for acutely modified genes following CVX-060 treatment in a responsive XG model. Additionally we compiled a list of all biomarkers reported to play a role in VEGF-inhibitor response, since the key predictive markers may be difficult to find from unbiased, whole-genome approaches on a small set of samples [50]. This large set of biomarkers was used to run a number of different correlation analyses with CVX-060 response in a focused XG panel. Finally, a second XG panel was employed to prospectively evaluate the predictive power of the findings. 

Whether the predictive markers identified here for CVX-060 will translate to clinic is unknown. Despite intensive preclinical and clinical analyses over the past decade with VEGF/VEGFR-targeting agents very few markers have correlated with efficacy, and contradicting findings have also been reported [2-4]. This may be a phenomenon of VEGF-axis therapies or anti-angiogenic therapies in general. Angiogenesis is a complex and dynamic process, with numerous growth factors and receptors involved, suggesting that efficacy of non-VEGF targeting agents (particularly those with different roles) might be governed by a unique set of markers. VEGFs role in angiogenesis is believed to be primarily in invasive capillary sprouting, while the angiopoietin family role is temporally and functionally different (vessel maturation/remodeling) [5-8]. In sum, identifying a simple predictive assay for anti-angiogenics may be a difficult task however there is hope for greater translational impact with angiopoietin biomarker work than has been seen with prior VEGF results. 

## Supporting Information

Figure S1 **Least Angle Regression (LAR) code used for identifying predictive biomarkers.** Full analysis settings and results are shown.
(PDF)

Figure S2 **Tumor inhibition curves in training set XG models.** Efficacy data used for table 1 TGI calculations is shown. Statistical difference between vehicle (PBS) and CVX-060 groups (*,**,*** = P <0.05, 0.01, or 0.001, respectively) determined by paired t-test.
(TIFF)

Table S1 **Data used for statistical analysis.** Tab 1: Tumor growth inhibition (TGI) achieved with CVX-060 treatment (10 mg/kg IP, QW until study termination) for each cell line xenograft (XG) or patient derived xenograft (PDX) model. Data represents median of 10 animals per XG. Training Set represents the 15 XG/PDX models used for building the biomarker hypothesis, Testing Set represents the 11 XG models used for prospective analysis of the hypothesis.
Tab 1: Median concentration of each biomarker in each XG model, as determined by ELISA of tumor lysate (500 mm3 size tumors). Values shown are normalized to total protein concentration (BCA) and are shown in ug/g, ng/g, or pg/g.
Tab 2: Number of individual tumors analyzed by ELISA for each XG and biomarker.
(XLSX)

Table S2 **Lack of correlation between TGI and common somatic mutations.** Common somatic mutations observed in cancer were queried in the XG models via comparison with COSMIC public database or via Sequenom OncoCarta v1.0 analysis of XG tumor lysate (500 mm^3^ tumors) and plotted against tumor growth inhibition (TGI). Blank box = no data available, wt = wild type.
(TIF)

Table S3 **Lack of correlation between TGI and anti-angiogenic related SNPs.** Haplotypes for 8 single nucleotide polymorphisms (SNPs) potentially related to anti-angiogenic therapeutic response were detected in tumor lysate (500 mm^3^ tumors) from the XGs by qPCR and plotted against tumor growth inhibition (TGI). The SNPs evaluated here have been previously reported as correlating with bevacizumab^37^ and/or pazopanib response in RCC^38^. Blank box = no data available.
(TIF)
