# Supplementary material for: Predictive Markers of Efficacy for an Angiopoietin-2 Targeting Therapeutic in Xenograft Models
Source: PLoS One. 2013 Nov 14;8(11):e80132. doi: 10.1371/journal.pone.0080132 (PMC3828186; doi:10.1371/journal.pone.0080132)
Supplement: Figure S1 — Least Angle Regression (LAR) code used for identifying predictive biomarkers. Full analysis settings and results are shown. (PDF) [file pone.0080132.s001.pdf]

Least Angle Regression for prediction of continuous response

Description of the problem:

Column of description sheet defining response variable: TGI
number of filtered genes 5
% error threhsold: 0
Algorithm used: LAR (least angle regression)
Inclusion of 2 way interaction: No
Cross-validation type: 10 fold
Column of the Experiment Descriptors sheet that defines training/prediction variable: Set

---

Prediction model

The prediction of new samples can be calculated by the formula: $\sum_i c_i x_i$ + 86.765 where c_i and x_i are the coefficient and gene expression for the i-th gene, respectively. The gene expression is the (normalized) log ratios for dual-channel data and log intensities for single-channel data. If two way interaction option is selected, each gene expression has to be subtracted by a constant defined below and x_i can be either an individual gene expression or a product of two gene expression.

		Coefficient
1	Angpt1	-2.159
2	EGF	-1.03
3	EMMPRIN	-5.822

Scatterplot of the actual response vs the predicted response from the cross-validation

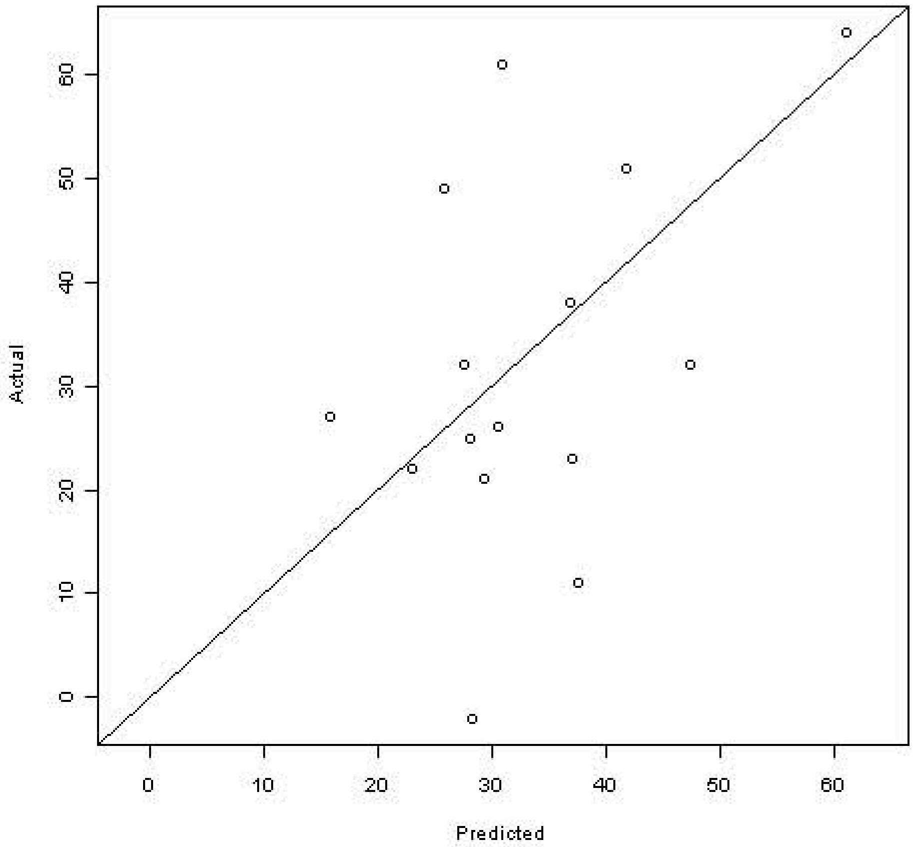

	Predicted	Actual
OVCAR5	28.286	-2
Caki1	30.503	26
ES2	37.609	11
HeyC2	29.354	21
HT29	15.761	27
A498	23.004	22
A2780	27.516	32
G401	47.455	32
IGROV1	37.019	23
SN12CCP	28.185	25
OVX276	36.915	38
OV90	41.868	51
colo205	61.221	64
SKOV3	25.835	49
OVX243	30.896	61

The R^2 for the model is: 0.19

10-fold cross-validated mean squared error curve

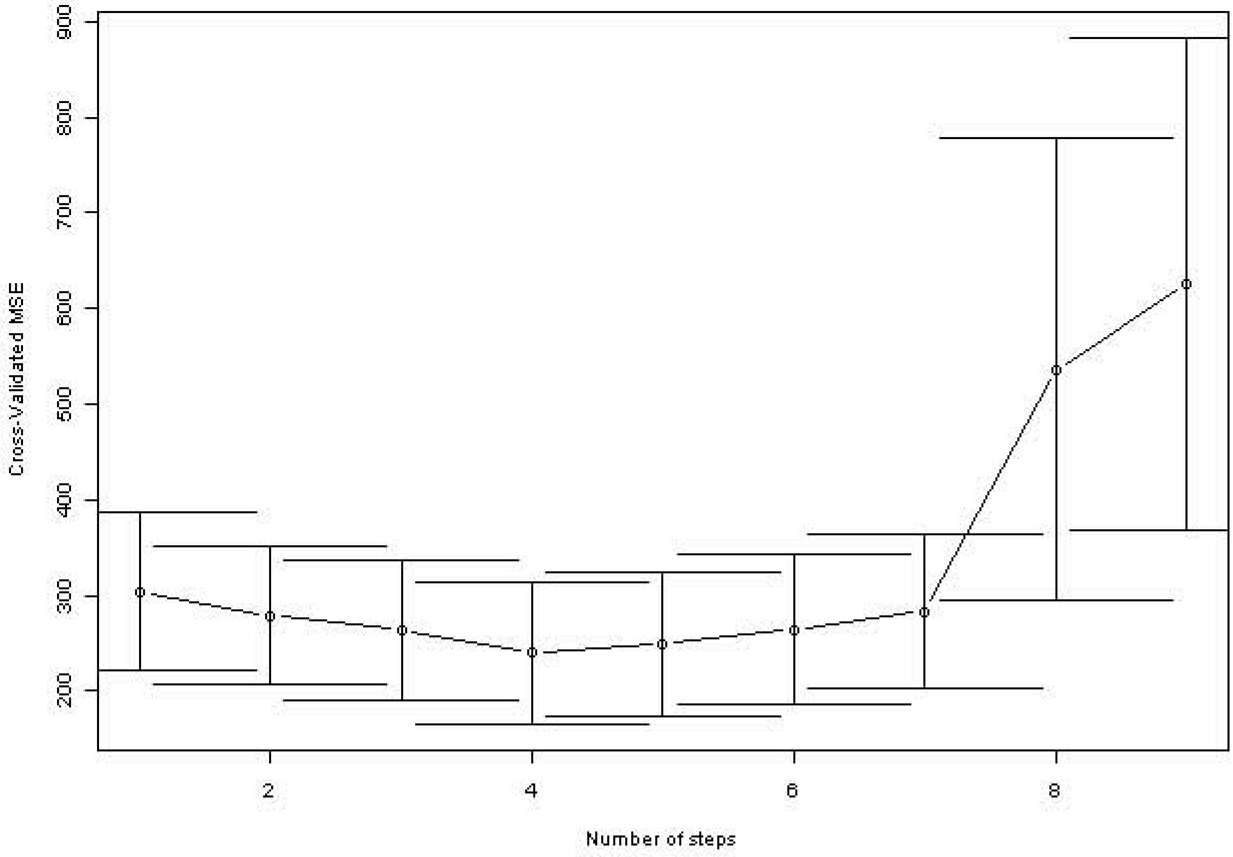

The x-axis stands for the algorithmic steps relative to the model containing n genes. The y-axis stands for a cross-validated estimate of the squared prediction error for each step. The standard error bands are included for the standard error of the CV curve at each step.

Click here to view expression of significant genes

Quantitative trait predictions of new samples:

Array id	Prediction
**A549**	49.709
**A431**	30.476
**MDA.MB435**	44.494
**U87.MG**	44.796
**D45MG**	27.521

**U251**	17.633
**NCIH441**	23.653
**SHP77**	31.415
**SKMEL1**	28.184
**NCI.H209**	30.294
**NCI.H720**	27.904

Filtering parameters:

R version 2.13.2 (2011-09-30)
Name of the project file: Project.xls
Time of the analysis: Tue Jun 12 12:48:43 2012

BRB-ArrayTools Version: 4.2.1 - Stable Release

- **Spot Filters: OFF**
- **Average the replicate spots within an array: OFF**
- **Normalization: OFF**
- **Gene Filters: OFF**
- **Gene Subsets: OFF**
