# Supplementary figures and images for: Predictive Markers of Efficacy for an Angiopoietin-2 Targeting Therapeutic in Xenograft Models
Source: PLoS One. 2013 Nov 14;8(11):e80132. doi: 10.1371/journal.pone.0080132 (PMC3828186; doi:10.1371/journal.pone.0080132)

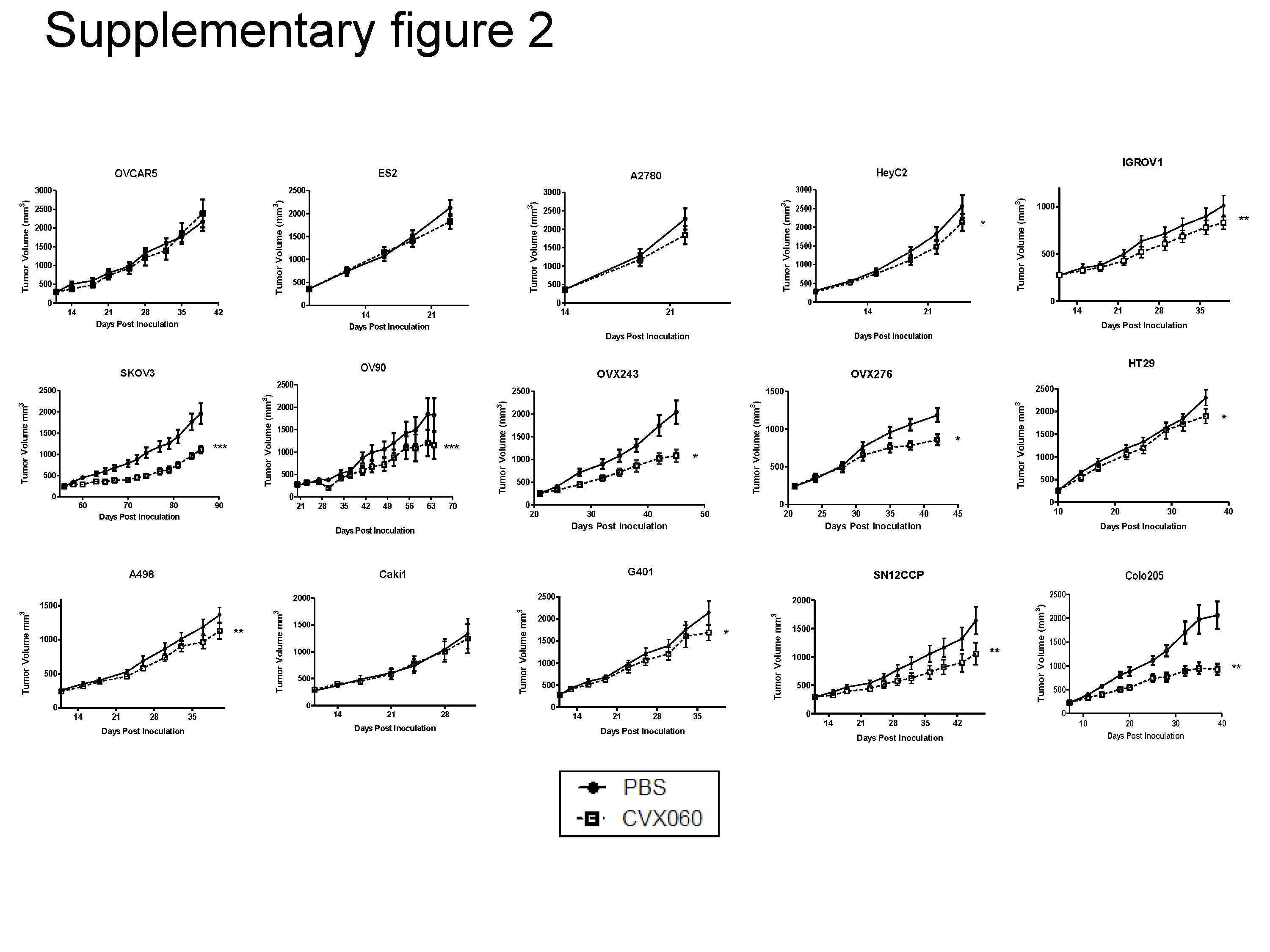

Supplement: Figure S2 — Tumor inhibition curves in training set XG models. Efficacy data used for table 1 TGI calculations is shown. Statistical difference between vehicle (PBS) and CVX-060 groups (*,**,*** = P <0.05, 0.01, or 0.001, respectively) determined by paired t-test. (TIFF) [file pone.0080132.s002.tiff]

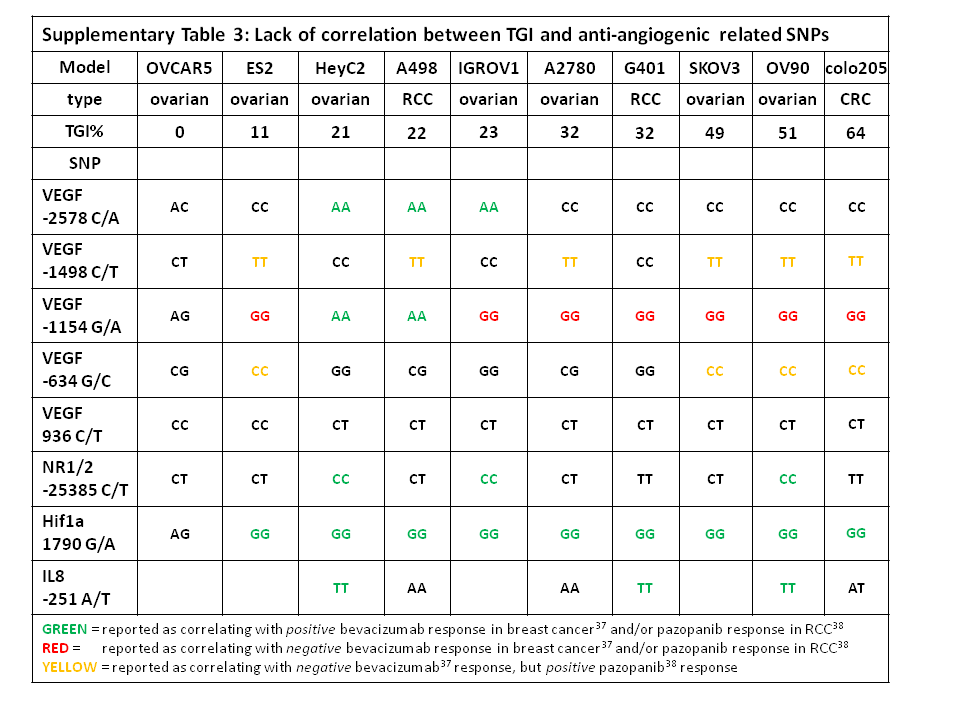

Supplement: Table S3 — Lack of correlation between TGI and anti-angiogenic related SNPs. Haplotypes for 8 single nucleotide polymorphisms (SNPs) potentially related to anti-angiogenic therapeutic response were detected in tumor lysate (500 mm3 tumors) from the XGs by qPCR and plotted against tumor growth inhibition (TGI). The SNPs evaluated here have been previously reported as correlating with bevacizumab37 and/or pazopanib response in RCC38. Blank box = no data available. (TIF) [file pone.0080132.s005.tif]
